# The Prognosis of Allocentric and Egocentric Neglect: Evidence from Clinical Scans

**DOI:** 10.1371/journal.pone.0047821

**Published:** 2012-11-01

**Authors:** Magdalena Chechlacz, Pia Rotshtein, Katherine L. Roberts, Wai-Ling Bickerton, Johnny K. L. Lau, Glyn W. Humphreys

**Affiliations:** 1 Department of Experimental Psychology, University of Oxford, Oxford, United Kingdom; 2 Behavioural Brain Sciences Centre, School of Psychology, University of Birmingham, Birmingham, United Kingdom; University of Cambridge, United Kingdom

## Abstract

We contrasted the neuroanatomical substrates of sub-acute and chronic visuospatial deficits associated with different aspects of unilateral neglect using computed tomography scans acquired as part of routine clinical diagnosis. Voxel-wise statistical analyses were conducted on a group of 160 stroke patients scanned at a sub-acute stage. Lesion-deficit relationships were assessed across the whole brain, separately for grey and white matter. We assessed lesions that were associated with behavioural performance (i) at a sub-acute stage (within 3 months of the stroke) and (ii) at a chronic stage (after 9 months post stroke). Allocentric and egocentric neglect symptoms at the sub-acute stage were associated with lesions to dissociated regions within the frontal lobe, amongst other regions. However the frontal lesions were not associated with neglect at the chronic stage. On the other hand, lesions in the angular gyrus were associated with persistent allocentric neglect. In contrast, lesions within the superior temporal gyrus extending into the supramarginal gyrus, as well as lesions within the basal ganglia and insula, were associated with persistent egocentric neglect. Damage within the temporo-parietal junction was associated with both types of neglect at the sub-acute stage and 9 months later. Furthermore, white matter disconnections resulting from damage along the superior longitudinal fasciculus were associated with both types of neglect and critically related to both sub-acute and chronic deficits. Finally, there was a significant difference in the lesion volume between patients who recovered from neglect and patients with chronic deficits. The findings presented provide evidence that (i) the lesion location and lesion size can be used to successfully predict the outcome of neglect based on clinical CT scans, (ii) lesion location alone can serve as a critical predictor for persistent neglect symptoms, (iii) wide spread lesions are associated with neglect symptoms at the sub-acute stage but only some of these are critical for predicting whether neglect will become a chronic disorder and (iv) the severity of behavioural symptoms can be a useful predictor of recovery in the absence of neuroimaging findings on clinical scans. We discuss the implications for understanding the symptoms of the neglect syndrome, the recovery of function and the use of clinical scans to predict outcome.

## Introduction

Persistent visuospatial deficits are often associated with overall poor functional outcome following stroke [1], [2]. The most common visuospatial disorder associated with stroke is unilateral neglect [3]. While neglect symptoms recover rapidly in some patients, in other cases the problems persist and contribute significantly to poor return to independent living [4], [5], [6]. It is thus important to delineate which lesions are associated with persistent neglect symptoms and which with recovery of function.

### Different forms of neglect

Unilateral neglect is diagnosed when patients fail to attend to stimuli presented on the side of space contralateral to their lesions [7]. However, unilateral neglect represents a complex syndrome with different patients showing a varied combination of impairments [1], [8]. Dissociable cognitive deficits within the neglect syndrome have now been reported both across a variety of different measures (e.g., line cancellation vs. bisection) and even within the same task [1], [9], [10]. Dissociations can be found between the presence of neglect symptoms in different modalities as well as between different sectors of space [8], [11], [12], [13], [14], [15]. Of most relevance to the current study is the dissociation between egocentric neglect, expressed through inattention to stimuli presented on the contralesional side of the body [16], [17], and allocentric neglect, shown by poor report of elements on the contralesional side of individual objects [16], [18], [19], [20], [21]. It is striking that egocentric and allocentric neglect can even be found on different sides of space within the same individual (e.g., [22], [23]). This contrasting patterns of spatial deficit within single cases makes it difficult to account for the dissociation in terms of a single gradient of deficit across space (cf. [24]). Rather the data fit with the notion that different visual representations are coded within the brain perhaps for different purposes (e.g., egocentric representations to help guide spatial exploration; allocentric representation for object recognition; see [25], for one explicit computational account).

### The neuroanatomy of neglect

There is also evidence that egocentric and allocentric neglect are associated with different brain lesions ([9], [14], [26], [27]; see below). For example, Chechlacz et al. [27] demonstrated that, after right hemisphere damage, left allocentric neglect is associated with lesions to the right posterior superior temporal sulcus, angular, middle temporal/inferior temporal and middle occipital gyri, while left egocentric neglect is linked to more right anterior lesions including the middle frontal, postcentral, supramarginal and superior temporal gyri and the insula. In contrast, damage to the right temporo-parietal junction is associated with both forms of neglect. Similar dissociations have been noted by several other groups (e.g., [9], [14], [26]). These contrasting lesion sites, linked to different neglect symptoms, may help to explain previous disparities in lesion-symptom mapping in the syndrome. Specifically, some groups have previously argued that the syndrome is linked to damage to the posterior parietal cortex, while others have reported damage within brain regions including the superior temporal gyrus, insula and basal ganglia (on the one hand see [28], [29], [30]; on the other see [31], [32], [33], [34]; see [91] for meta-analysis and overview).

In addition to the grey matter lesions associated with neglect there are also white matter lesions. Such lesions disrupt connectivity within attentional networks and this has led some researchers to regard neglect as a disconnection syndrome [35], [36]. Specifically, neglect has been reported following damage to the superior longitudinal (SLF; [27], [35], [37], [38], [39]), the inferior longitudinal (ILF; [27], [40], [41]) and the inferior fronto-occipital fasciculi (IFOF; [27], [37], [40], [42]). Interestingly, Chechlacz et al. [27] found that damage within long association pathways including the right ILF, the IFOF and the SLF, were linked to both allocentric and egocentric neglect. They suggested that the different representations of space, formed in different cortical regions, were connected to anterior, action control areas of the brain through common white matter tracts.

### Recovery of function

The recovery rates from unilateral neglect following stroke vary between reports but roughly about one third of patients show persistent visuospatial problems several months after stroke [2], [4], [5]. It has been postulated that several different factors might have a significant impact on neglect recovery including the initial severity of the deficit(s), the presence of visual field defects, age and age-associated brain atrophy as well as lesion size and location [4], [43], [44], [45], [46], [47], [48], [49], [80]. Several studies indicate that neglect recovery can be predicted from neuroanatomical data [45], [48], [50], [51], [52]. For example Maguire and Ogden [51] have shown that persistent neglect is associated with lesions that involve at least three cortical lobes as well as the basal ganglia but that parietal lesions *per se* are not essential for chronic neglect. Karnath et al. [50] provide additional evidence that lesions within the temporal cortex (including the superior and middle temporal gyri) and basal ganglia play a critical role for predicting chronic neglect. Recovery can also be linked to white matter damage. Samuelsson et al. [48] reported that chronic neglect was highly correlated with damage to paraventricular white matter within the temporal lobe while Karnath et al. [50] linked damage to the inferior fronto-occipital (IFOF) and uncinate fasciculi to chronic as well as acute neglect.

While these studies clearly suggest significant relationships between the location of brain damage and the recovery of neglect post-stroke, none of these reports takes into account the heterogeneity of neglect deficits in relation to the presence of a spatial disorder in chronic cases. One step towards this was recently reported by Kurshid et al. [53] who noted that reperfusion of contrasting cortical areas can also predict recovery of different neglect symptoms in the acute stage after stroke – for example, they found that reperfusion of ventro occipito-temporal regions 3–5 days post lesion was linked to improvements in allocentric neglect while reperfusion of more dorsal fronto-parietal areas was associated with improvements in egocentric neglect. Furthermore, another recent study by Saj et al. [52] points to the importance of the type of tests used for neglect diagnosis when examining recovery. The authors conclude that investigations which do not distinguish different cognitive components of the syndrome may hamper understanding not only of the neural substrates of neglect but also the mechanisms of recovery [52]. Here we assessed for the first time the neuroanatomical correlates of sub-acute vs. persistent visuospatial deficits associated with two distinct aspects of the neglect syndrome – egocentric and allocentric neglect (cf. [9], [13], [26], [27].

Interestingly, in an analysis of a large-scale screen of stroke patients using the Apples test that we employ, Bickerton et al. [10] noted that impairments in allocentric neglect are predictive of poor functional outcome in patients (e.g., on the Barthel index), while this was not necessarily the case for egocentric neglect. In addition the two forms of neglect correlated with different behavioural impairments (allocentric neglect with aspects of gesture reproduction, egocentric neglect with performance on multi-step tasks), highlighting the need to distinguish the different spatial impairments when attempting to predict outcome from lesion data. The analyses we conduct here also controlled for potential confounding factors such as aetiology (the type of stroke: ischemia or hemorrhage), age-related changes, time from stroke to scan, lesion volume and the patient's overall orientation and anosognosia. This enabled us to examine the neuronal substrates of different neglect symptoms at sub-acute and chronic stages, with effects of other factors, which may co-vary with recovery, accounted for.

The current study used clinical scans derived as part of the routine clinical care for patients. This has a direct translational implication, if lesion site detected through such scans is informative about patient prognosis. We employed whole brain statistical analyses (voxel-based morphometry VBM; [60]) to evaluate common structure-function relationships across the whole brain, separately for grey and white matter. The analysis was performed on computed tomography (CT) scans and treated the behavioural measurements as continuous variables rather than as categorical scores, which increased both the ability to tease apart the different types of neglect and the sensitivity for detecting brain-behaviour associations. We discuss the implications of the results for understanding the symptoms of neglect, the recovery of function and the use of clinical scans to predict outcome.

## Methods

### Participants

All patients were recruited as part of the BUCS project (Birmingham University Cognitive Screen, http://www.bucs.bham.ac.uk) from participating stroke units across the West Midlands area (United Kingdom). We excluded from the study patients who either had enlarged ventricles or poor quality CT scans in order to prevent artifacts in the neuroimaging analyses. A total of 160 stroke patients (92 males and 68 females; average age of 68.7 years, range 31 to 91 years; see Table 1 for full demographic and clinical data) were included. Within this group 73 patients had lesions within the territory of the middle cerebral artery (MCA) and 21 patients had lesions within the territory of the posterior cerebral artery (PCA). The analysis was performed both on patients who suffered ischemic stroke (144 patients) and patients with hemorrhagic stroke (16 patients). Behavioural data were only collected from patients who were physically stable, willing to perform the task and had a concentration span of at least ∼60 minutes (judged clinically). Clinical and demographic data were obtained from the patients' clinical files. All participants provided written informed consent in agreement with ethics protocols approved by the National Research Ethics Service: Essex 1 Ethics Committee.

**Table 1:** Patient details: clinical and demographic data.

	Neglect*** (n = 55)		No neglect (n = 105)	
	Sub-acute	Chronic	Sub-acute	Chronic
Age in years (mean/SD)	69.3/12.3	N/A	68.3/12.7	N/A
Sex (M/F)	29/26	N/A	63/42	N/A
Aetiology (ISCH/BL)	47/8	N/A	97/8	N/A
Lesion size (mean/SD)*	62.0/87.4 cm^3^	N/A	40.6/65.6 cm^3^	N/A
Handedness (R/L)	49/6	N/A	92/13	N/A
Scan time since stroke days (mean/SD)	5.5/12.9	N/A	3.2/7.7	N/A
BUCS** in days (mean/SD)	28.5/21.5	280.3/14.1	21.3/17.3	282.1/16.3
Orient1 mean/SD (max/range)	7.5/1.3 (8/1–8)	7.8/0.6 (8/7–8)	7.6/1.2 (8/3–8)	7.8/0.9 (8/5–8)
Orient2 mean/SD (max/range)	5.4/1.0 (6/2–6)	5.6/0.9 (6/1–6)	5.7/0.7 (6/3–6)	5.9/0.3 (6/4–6)
Nosognosia (Orient3) mean/SD (max/range)	2.8/0.5 (3/0–3)	3.0/0.2 (3/2–3)	2.9/0.3 (3/1–3)	3.0/0.2 (3/2–3)
Left VE (uni asymmetry) mean/SD (max/range)	0.5/1.2 (4/0–4)	0.2/0.8(4/0–4)	0.1/0.4 (4/0–4)	0/0 (4/0)
Right VE (uni asymmetry) mean/SD (max/range)	0.4/1.1 (4/0–4)	0.3/1.0/(4/0–4)	0.1/0.7 (4/0–4)	0.1/0.7 (4/0–4)
Left VE (bilat asymmetry) mean/SD (max/range)	2.0/3.0 (8/0–8)	1.1/2.3 (8/0–8)	0.1/0.8 (8/0–8)	0.1/0.2 (8/0–1)
Right VE (bilat asymmetry) mean/SD (max/range)	0.7/2.2 (8/0–8)	0.6/2.1 (8/0–8)	0.3/1.4 (8/0–8)	0.2/1.0 (8/0–8)
ACT accuracy mean/SD (max/range)	28.4/14.7 (50/1–49)	38.2/11.1 (50/15–49)	47.4/4.4 (50/35–50)	46.9/4.0 (50/31–50)
ACT/AFA (left deficits) mean/SD (max/range)	4.8/6.0 (25/0–20)	2.8/4.4 (25/0–20)	0.4/0.7 (25/0–3)	0.5/1.0 (25/0–4)
ACT/AFA (right deficits) mean/SD (max/range)	1.0/2.2 (25/0–10)	1.3/3.2(25/0–14)	0.4/0.8 (25/0–4)	0.4/0.8 (25/0–2)
ACT/AIncA (left deficits) mean/SD (max/range)	3.1/5.1 (50/0–19)	1.3/3.0 (50/0–17)	0.1/0.2 (50/0–1)	0.1/0.4 (50/0–2)
ACT/AIncA (right deficits)mean/SD (max/range)	1.0/2.0 (50/0–11)	0.5/1.8 (50/0–6)	0.1/0.3 (50/0–1)	0.2/0.6(50/0–3)

* Overall lesion size (volume) in the neglect group was not significantly larger than in the group without neglect symptoms (t(158) = 1.7, p>0.5);

** For the acute/subacute phase the number of days indicate stroke to test (initial BUCS) interval and at the chronic phase number of days indicate the interval between initial BUCS test and follow up BUCS;

*** Patient who at sub-acute phase following stroke showed any type of neglect symptoms including egocentric and allocentric neglect for either left or right side of space; ACT, Apple Cancellation task; the maximum achievable score in the Apple Cancellation task is 50 (ACT accuracy). The cut-off for total numbers of target (full apples) omissions i.e. accuracy score is 40/50. Egocentric neglect is determined by whether patients miss targets (complete apples) on the left or right side of the page (asymmetry score calculated based on left- vs. right-side errors, ACT/AFA asymmetry score for full apples indicating either left or right deficits). Allocentric neglect is determined by whether patients make false positive responses by cancelling incomplete apples (distractors) where the gap is on either the right or left side of each apple, irrespective of the position of the (incomplete) apple on the page (asymmetry score calculated based on left- vs. right-side errors, AIncA asymmetry score for incomplete apples); BL, bleed/hemorrhagic stroke; F, female; ISCH, ischemic stroke L, left; M, male; max/range, maximum achievable score and range of scores within the group of patients; Orient1, orientation measure assessing personal information; Orient2, orientation measure assessing time and space awareness; R, right; SD; standard deviation; VE, visual extinction test, the task consists of 4 unilateral left, 4 unilateral right and 8 bilateral trials, asymmetry score calculated based on left- vs. right-side misses.

### Behavioural measures

#### Cognitive profile

The initial neuropsychological testing took place in the sub-acute phase following stroke onset and the average stroke to test interval was 24 days (±19.5; range 2–68 days; with 95% of patients being tested within two months and 78% of patients being tested within 1 month). The follow up neuropsychological testing was carried out at the chronic phase approximately 9 months following initial testing with the average test to test interval of 281 days (±15.6). The cognitive profile of each patient was derived using the BCoS, a test instrument developed to screen patients for a range of cognitive problems following stroke onset [81]. The BCoS is aphasia and neglect-friendly and within 1 hour provides assessment based on 23 tests within 5 broad cognitive domains: Attention and Executive functions, Memory, Language, Praxis/Control and Planning of Action, and Mathematical/Number abilities. For the sub-acute tests, the BCoS was administered in hospital settings and at follow-up it was administered either in the hospital, a rehabilitation clinic, the School of Psychology, Birmingham University or during a home visit. All examiners were blind to the location of the stroke and the patient's condition. In this study we were interested in visuospatial attention deficits and based our analysis on 2 sub-tests: Apple cancellation (measuring different forms of neglect) and Visual Extinction (see below for details). BCoS also provides an assessment of the patient's awareness of their general setting and circumstance (the orientation questions that assess knowledge of personal information, awareness of time, place, medical condition and anosognosia). Three orientations measures derived from BCoS were used in the current study: 1) eight questions regarding knowledge of personal information (e.g. ‘what is your first name?’); 2) six questions on the orientation of the patient in time and space (e.g.‘where are you right now?’); 3) three questions measuring the awareness to the medical condition and one owns body (‘can you show me your left/right hand’; ‘why are you here?’; “do you have any problems moving your arms or legs”). The orientation measures were used in the analyses to control for overall comprehension and for presence of anosognosia and somatoparaphrenia often associated with neglect symptoms (e.g. [54], [55], [56]).

#### Neglect assessment

**Figure 1:**
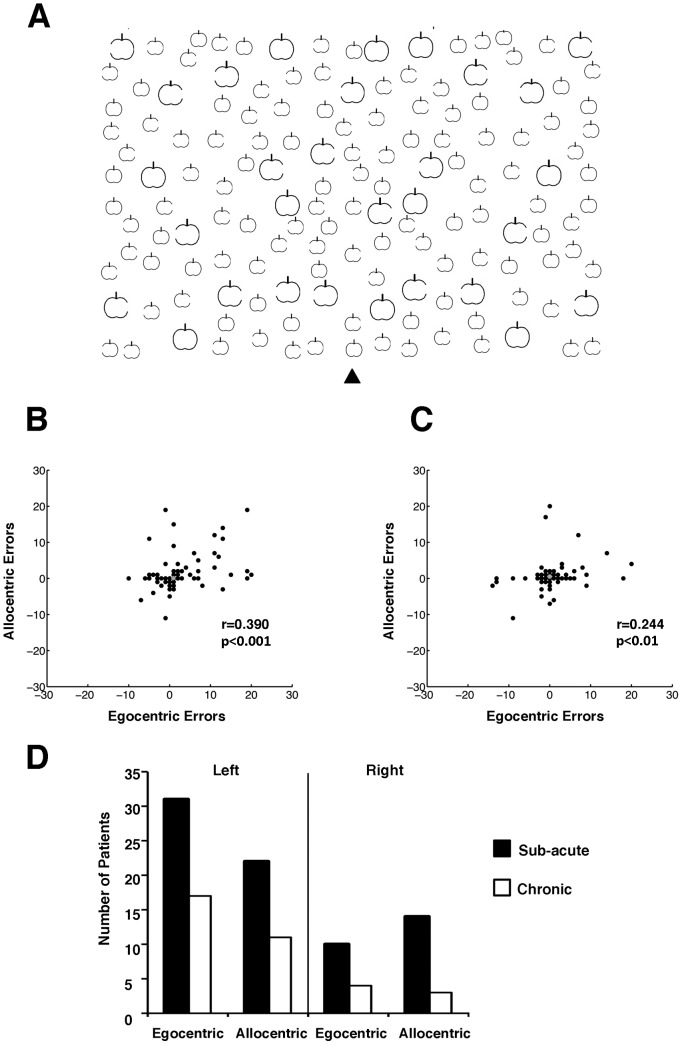
Example of the Apples cancellation task (A) used to simultaneously test for allocentric and egocentric symptoms. In this test patients are asked to cross out all the full apples. Egocentric neglect is measured by whether patients miss targets (full apples) predominantly on one side of the page and allocentric neglect is measured by whether patients make false positive responses by cancelling predominantly left or right distracters (according to the position of the gap defining a distracter; for full details and scoring see the Methods section). (**B**) and (**C**) present scatterplots of egocentric neglect errors against allocentric neglect errors at the sub-acute (**B**) and chronic (**C**) phases following stroke. There was significant correlation between allocentric and egocentric neglect scores at either phase. Please note that the middle grey dot corresponds to results for non-impaired patients. (**D**) Behavioural results – the number of patients with egocentric and allocentric neglect symptoms at the sub-acute and chronic phase following stroke.

Neglect was assessed using the Apple Cancellation task [10], [27]. Bickerton et al. [10] provide data validating the Apple Cancellation Test in relation to other standardized tests of neglect, as well as they also report on a comparison between the Apple Cancellation task and other neglect measures and discuss the relations between Apple Cancellation and tests of other cognitive processes, everyday activities and affect. This cancellation task is similar to the gap detection task by Ota et al. [57] and is designed to simultaneously measure egocentric and allocentric neglect. Participants are presented with a page (A4) in landscape orientation with 50apples presented across 5 invisible columns, one middle, one near left, one far left, one near right and one far right. Each column contained 10 complete apples (targets) along with distractors; the distractors were apples with either a left or a right part missing (incomplete apples; Figure 1A). Egocentric neglect is measured by whether patients miss targets (complete apples) on one side of the page. Allocentric neglect is measured by whether patients make false positive responses by cancelling distractors (i.e. incomplete apples) whose gap was on the left or right of the shape. In the neuroimaging analyses we used asymmetry scores for left and right allocentric (e.g., false alarms to distractors with a gap on the left – false alarms to distractors with a gap on the right) as well as normalized asymmetry scores for left and right egocentric neglect from the Apple Cancellation task (see [10], [27], [81] for details and additional information about this test). The cut off scores for neglect, derived from performance of a group of 100 control participants age-matched to the stroke population, were as follows: egocentric neglect - asymmetry for full apples <−2 right side errors or >3 left side errors; total numbers of target omissions i.e. accuracy score 40/50; allocentric neglect - asymmetry for incomplete apples (based on <2.5th percentile) <−1 right side errors or >1 left side errors. The cut-off for the total number of target omissions was 40/50; based on <2.5th percentile for patient performance [27].

#### Visual extinction

The task consisted of 4 unilateral left, 4 unilateral right and 8 bilateral trials. Testing for visual extinction was done by the examiner raising his/her left and right index fingers on either side of his/her head and then moving (two brief bending movements) either left or right (unilateral trials) or both fingers simultaneously (bilateral trials). For each patient we calculated left and right asymmetry scores on two item trials and on unilateral trials. We also calculated the left and right extinction index. The difference in the asymmetry scores on bilateral vs. unilateral trials was assessed, to index any spatially selective drop in response to two stimuli relative to the response to one stimulus. This was done separately for both left- and right-side items. To do this we calculated an extinction index i.e. the unilateral asymmetry score multiplied by two minus the bilateral asymmetry score, taking into account the difference in the number of trials. The extinction index and the asymmetry score for both left- and right-side unilateral items were entered into the statistical models.

Each patient's behavioural performance was classified based on cut-offs drawn from the BCoS. Patients were classed as having a clinical deficit on measures of visual extinction if their scores on the task fell outside the control norms taken from 70 healthy controls without history of brain lesion or any neurological disorders. The cut off scores for visual extinction task are as follows: unilateral trials (both left and right) <4 impaired; left bilateral trials <7 impaired; right bilateral participants younger than 74 years old <8 impaired and participants older than 75 years old <7 impaired.

### Neuroimaging assessment

Computed Tomography (CT) scans were acquired for all patients as part of their routine clinical assessment following stroke and hospital admission. The average time between suspected stroke and the CT scan was 3.9 days (±10.2, with 93% of cases within a week). Out of 160 patients, 43 had no visible lesion on CT scan (excluding atrophy and non-specific white matter changes) and 27 of these had scans that were obtained less than 4 days following stroke, including 17 patients with scans taken within first 24 h following stroke. Out of these 43 patients with no visible lesion, 18 patients had neglect symptoms (3 left allocentric, 5 left egocentric, 5 right egocentric and 5 right allocentric neglect; see also Figure S2). We note here that all 160 segmented CT scans were entered into all statistical models (including these with no visible lesions). It may be the case that, within the group of patients with no visible lesions, some had sub-threshold changes in the grey/white matter densities, which although not visible to the eye, were detected by VBM analyses.

The neuroimaging data were acquired using the following scanners: Siemens Sensation 16, GE Medical System LightSpeed 16 and LightSpeed Plus. The images covered the whole brain with an in-plane resolution of 0.5×0.5 mm^2^ and a slice thickness varying between 4–5 mm.

### Neuroimaging analysis

#### Image preprocessing

Before the preprocessing stage, the quality of all CT scans was assessed by eye and all bad quality data sets (head movement or other image artefact) were removed. Subsequently, the remaining CT images were pre-processed using SPM8 (Statistical Parametric Mapping, Welcome Department of Cognitive Neurology, London UK). The images were first normalized to an in-house CT template [82]. The normalization was predominantly based on skull shape and was designed to transform the images into MNI space. In the next step we used the unified segmentation algorithm as implemented in SPM8 [58]. In this unified model, the tissue class priors are encoded by de-formable tissue probability maps. The a-priori tissue class maps indicate the probability of finding expected signal sources of grey matter (GM), white matter (WM), cerebrospinal fluid (CSF), fat, bone and air (i.e. six different tissues classes), at each voxel of the image. As the CT scans were acquired following stroke, to account for the presence of an abnormal tissue associated with stroke, we adapted here a similar approach to [59] and included additional, seventh tissue class. Specifically, in the additional probability map we assumed that in each grey or white matter voxel there was a 10% chance of it having a different intensity and thus representing an abnormal tissue class. In addition, we constrained the classification of GM and WM to each being based on a single Gaussian (normal) distribution, while two Gaussian distributions were used to model the intensities in the abnormal tissue class. This later procedure was used to account for any possible in-homogeneity of the abnormal tissue. CT as opposed to MR images do not suffer from field bias due to field strength inhomogeneity, therefore we did not correct for that during pre-processing. In the final step of image pre-processing the segmented GM and WM images were smoothed with a 12-mm FWHM Gaussian filter to accommodate the assumption of random field theory used in the statistical analysis [83]. Finally, the quality of the segmentation and normalization procedures was assessed for each patient and images where the segmentation failed were removed from the analyses. The pre-processed GM and WM images were further used in the analyses to determine voxel-by voxel relationships between brain damage and visuospatial deficits (see below).

See Figure S1 for examples of output of the modified unified segmentation of patients' CT scans.

#### Lesion volume and lesion overlap map

The lesion of each patient included in the current study was semi-automatically identified using the modified unified segmentation (as described above) and also a voxel-based outlier detection procedure based on general linear model (see Methods S1 for a full description). Outlier maps were generated that coded the degree of abnormality of each voxel (based on the comparison to the normal range from 105 control scans). The outlier maps were then thresholded into binary lesion maps for each individual patient. The binary lesion maps were overlaid (summed across all patients using Image calculator function within SPM8). The lesion overlay map was created to represent the spatial distribution of lesions in our group of 160 patients (see Figure S3). For examples of lesion reconstruction for individual patients see Figure S4. The lesion volume for each patient was calculated using Matlab 7.8 (The MathWorks, Natick, MA, USA) based on individual lesions from automated lesion identification procedure. The estimated lesion volumes of all individual patients were used as covariates in the statistical models in VBM analyses (see below). All statistical comparisons evaluating the effect of lesion volume on the presence of neglect symptoms and neglect recovery were carried out using Matlab 7.8.

#### Voxel-based morphometry (VBM)

We applied random effects analyses within the general linear model framework [84] to compute correlations between the behavioral measures of visuospatial deficits at both (i) sub-acute (Analysis 1) and (ii) chronic (Analysis 2) phases post stroke in relation to the tissue damaged [60]. We used the full factorial design to generate models for GM and WM separately. The statistical models for Analyses 1 and 2 included the continuous scores for both left and right allocentric and egocentric errors (extracted from the Apple Cancellation task) as assessed at the acute and chronic phases post stroke respectively. This ensured that we could control and formally test for common and dissociated neuronal substrates that contribute to the two types of neglect being assessed. In all statistical models we also included four behavioural measures of other visuospatial problems: left and right asymmetry scores on unilateral trials and left and right visual extinction indices (extracted from the BCoS extinction test). This enabled us to examine the neuronal substrates of neglect symptoms with effects of other visuospatial deficits, which may co-vary with neglect, eliminated. All patients in the current study were recruited from BUCS project based on BCoS instrument, which do not include a measure of visual field defects *per se*. Therefore, the left and right unilateral asymmetry scores (i.e. left vs right unilateral misses) derived from the visual extinction test were included in all statistical models to reduce the spurious effects of the presence of visual field impairments. Additionally, to control for potential confounding factors in all statistical models we included as covariates age, gender, handedness, time from stroke to neuropsychological testing (or time of BUCS follow up for Analysis 2), time from stroke to scan, the type of stroke (ischemia or hemorrhage), lesion volume and 3 orientation measures assessing the patient's awareness of their general setting and circumstance (as described above). Our Results and Discussion sections focused on left neglect symptoms since, as shown by our behavioural data, these symptoms were more frequent and more severe than the neglect symptoms after right hemisphere damage (see Table 1; this is in agreement with previous reports, for a review see [8]; furthermore we found no significant relation between brain damage and either right allocentric or right egocentric neglect). However, we have not restricted our study to right hemisphere-lesioned patients and all statistical models included both left and right deficit scores (i.e. four separate covariates represented by continuous behavioural scores for left allocentric, right allocentric, left egocentric and right egocentric neglect). This was done to avoid biasing the results – for example, the exclusion of patients with left-hemisphere lesions could limit inferences about any potential contributions of the affected brain regions to both left and right deficits.

The dissociation between left allocentric and left egocentric neglect was assessed by using exclusive masking, while common brain regions were tested using conjunction analysis [61]. Using the exclusive mask allowed us to identify damaged areas involved in left allocentric but not left egocentric neglect and vice versa (at the voxel level the threshold for the exclusive masking was p<0.05 uncorrected). To further verify the dissociations between allocentric and egocentric neglect, we report in the tables the results (F-tests) of the interaction between allocentric and egocentric neglect regressors. Common mechanisms were tested using conjunction analyses [61] to highlight changes in voxel intensity that correlated with both left egocentric and left allocentric neglect. We discuss only those results where there was a significant effect at p<0.001 cluster-level corrected for multiple comparisons. For t-test we used a voxel amplitude of Z>3.5 and an extent threshold of 100 voxels and for the conjunction analysis we used Z>3 and minimal cluster size of 100 voxels. The brain coordinates are presented in standardized MNI space. The anatomical localization of the lesion sites within the grey matter was based on the Anatomical Automatic Labeling toolbox (AAL toolbox, [62], the Duvernoy Human Brain Atlas [63] and the Woolsey Brain Atlas (Woolsey et al., 2008). In order to localize white matter lesions associated with visual extinction in relation to specific white matter pathways we used the JHU White matter tractography atlas [64] and the MRI Atlas of Human White Matter [65].

## Results

### Behavioural findings

Table 1 presents the demographic and clinical data for all the patients, including performance on the Apple Cancellation Task at both the sub-acute and chronic phases following stroke. Table 2 contrasts the demographic and clinical details of the patients with only sub-acute neglect symptoms who recovered by 9 months and those with persistent (chronic) neglect symptoms, it further highlights the distribution of neglect symptoms within these two groups.

**Table 2:** Clinical and demographic details of patients with sub-acute and chronic neglect.

	Sub-acute neglect only* (n = 29)	Chronic neglect** (n = 26)
Age in years (mean/SD)	67.2/13.1	74.0/11.1
Sex (M/F)	16/13	13/13
Aetiology (ISCH/BL)	24/5	23/3
Lesion size (mean/SD)	29.2/52.4 cm^3^	104.9/105.0 cm^3^
Handedness (R/L)	23/6	26/0
ACT accuracy (mean/SD)	33.5/14.3	21.8/12.4
**Left allocentric neglect**		
number†	11	11
score (mean/SD)‡	5.7/4.3 (2.1/3.8)	10.0/6.7 (4.2/6.4)
**Left egocentric neglect**		
number†	14	17
score (mean/SD)‡	4.9/5.9(3.3/5.3)	9.3/5.5(6.9/6.2)
**Right allocentric neglect**		
number†	11	3
score (mean/SD)‡	3.5/2.8 (1.2/2.3)	3.5/1.7 (0.6/1.5)
**Right egocentric neglect**		
number†	6	4
score (mean/SD)‡	2.9/1.9(0.7/1.5)	4.6/3.2(1.4/2.7)

* Patient who at sub-acute phase following stroke showed any type of neglect symptoms including egocentric and allocentric neglect for either left or right side of space and who recovered completely by 9 months;

** Patient who at chronic phase following stroke showed any type of neglect symptoms, i.e. patients who did not recovered; † this refers to the total number of patients with specific neglect symptoms including these with one or both types of symptoms, see results section for details; ‡, average score across patients with specific deficit (in brackets mean/SD for the entire group); ACT, Apples Cancellation Task (see Methods section for full details).

#### Behavioural results at the sub-acute stage

The patients were classified on the basis of the control data. Out of the 160 patients included in the current study, 15 patients at the sub-acute phase (<3 months) showed both left egocentric and left allocentric neglect, and 3 showed both right egocentric and right allocentric neglect with varied severity of impairments (assessed relative to control performance based on the Apple Cancellation Task). Interestingly, 3 patients showed left egocentric and right allocentric neglect, and 1 patient showed right egocentric and left allocentric neglect (see [22], [23], [66] for previous reports on the occurrence of allocentric and egocentric neglect on opposite sides of space within single patients). Furthermore, 13 patients exhibited only left and 6 only right egocentric neglect; 6 patients exhibited only left allocentric neglect and 8 only right allocentric neglect (Figure 1D). Finally, 18 patients showed left visual extinction (4 of whom did not exhibit neglect), and 7 showed right visual extinction (6 of whom showed no neglect). Our data are in direct agreement with previously reported results on the frequency of neglect (e.g., Becker and Karnath, 2007; Eschenbeck et al., 2010). Specifically, Eschenbeck reported 32.4% or 25% depending on the types of diagnosis used (standard neglect battery or daily living activities) as the frequency of sub-acute neglect symptoms following right hemisphere strokes. Becker and Karnath (2007) reported that 24.3% of acute patients with right hemisphere strokes and 4.9% of patients with left hemisphere stroke had neglect symptoms. In our group 33.3% and 23.8% of patients had egocentric and allocentric neglect respectively following right hemisphere strokes, while 3.6% and 10.9% of patients had egocentric and allocentric neglect respectively following left hemisphere strokes (see Figure S2). Interestingly, a recent study (Khurshid et al., 2012) has found that, similar to our study, allocentric can be more frequent than egocentric neglect following left hemisphere damage (see also Bickerton et al., 2011).

Subsequently, based on the behavioural findings and in agreement with previous reports, we restricted our analyses to left neglect symptoms but all statistical models included as additional regressors right egocentric and right allocentric errors as well as left and right visual extinction scores, to avoid biasing the results based on priori assumptions with regards to the neuroanatomy of the syndrome and to control for additional visuospatial problems associated with left neglect (see Methods section). Figure S2 illustrates the frequency of allocentric and egocentric neglect after left and right hemisphere damage; please note that neglect was diagnosed in a small percentage of patients with no visible lesions on computed tomography scans.

#### Behavioural results at the chronic phase

Out of the 15 patients who showed both left egocentric and left allocentric neglect at the sub-acute phase, 7 patients persisted in showing both deficits at the chronic phase while 7 patients recovered from both symptoms and 1 patient recovered from egocentric but not from allocentric neglect. Out of 3 patients with both right egocentric and right allocentric neglect, 2 persisted in showing symptoms while 1 recovered. Furthermore, out of 13 patients who exhibited only left egocentric neglect at the sub-acute phase, 9 persisted in showing egocentric symptoms, while 4 recovered. Out of 6 patients with only right egocentric neglect, 2 persisted in showing symptoms and 4 recovered. Out of the 6 patients who exhibited only left allocentric symptoms, 2 persisted in showing allocentric symptoms, while 4 recovered. Out of 8 patients with only 8 right allocentric symptoms, only one did not recover. Finally, of the 3 patients who showed left egocentric and right allocentric neglect at the sub-acute phase, 2 recovered from both symptoms and 1 persisted with only left egocentric symptoms. The patient who exhibited right egocentric and left allocentric neglect at a sub-acute phase recovered from egocentric but not the allocentric symptoms (Figure 1D).

Note that in the all analyses we used continuous scores for both types of neglect symptoms. By accounting for the severity of the symptoms and not just for their categorical presence, we attempted to provide a sensitive assessment of the relations between the two types of neglect. Using these continuous scores we could test for correlations between the severity of allocentric and egocentric neglect at both the sub-acute and chronic phases following stroke. Interestingly, there was significant correlation between these two types of neglect at both the sub-acute (r = 0.390 at p<0.001: Figure 1B) and the chronic phase (r = 0.244 at p<0.01; Figure 1C), supporting a dissociative account of the syndrome (see also [10], [13]).

### Grey matter: Acute vs. chronic prognosis of allocentric vs. egocentric symptoms

**Figure 2:**
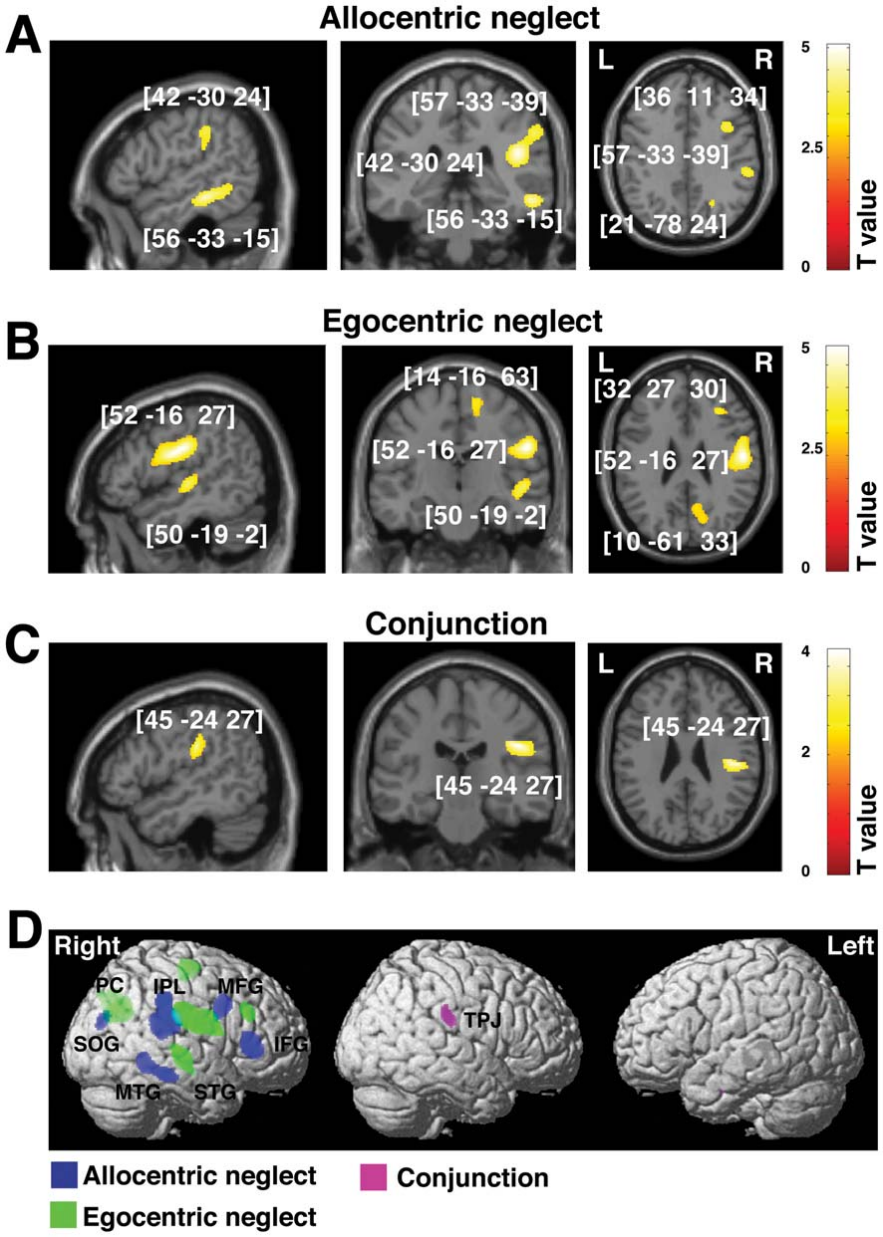
Voxel-wise statistical analysis of grey matter damage: allocentric vs. egocentric neglect at the sub-cacute phase following stroke. VBM results showing voxels corresponding to grey matter damage in (**A**) left allocentric, (**B**) left egocentric and (**C**) both forms of neglect (conjunction analysis). Please note that in **A**, **B** and **C** the lesioned areas are coloured according to their significance level in the VBM analysis, where a brighter colour means a higher t-value. The numbers in brackets indicate peak MNI coordinates. (**D**) To further illustrate the relationship between grey matter loss associated with allocentric and egocentric symptoms at the sub-acute phase, all clusters identified by VBM as de scribed above are plotted on a rendered brain. IFG, inferior frontal gyrus; IPL, inferior parietal lobule; MFG, frontal gyrus; MTG, middle temporal gyrus; PC, precuneus; STG, superior temporal gyrus; SOG, superior occipital gyrus; TPJ, temporal-parietal junction.

**Figure 3:**
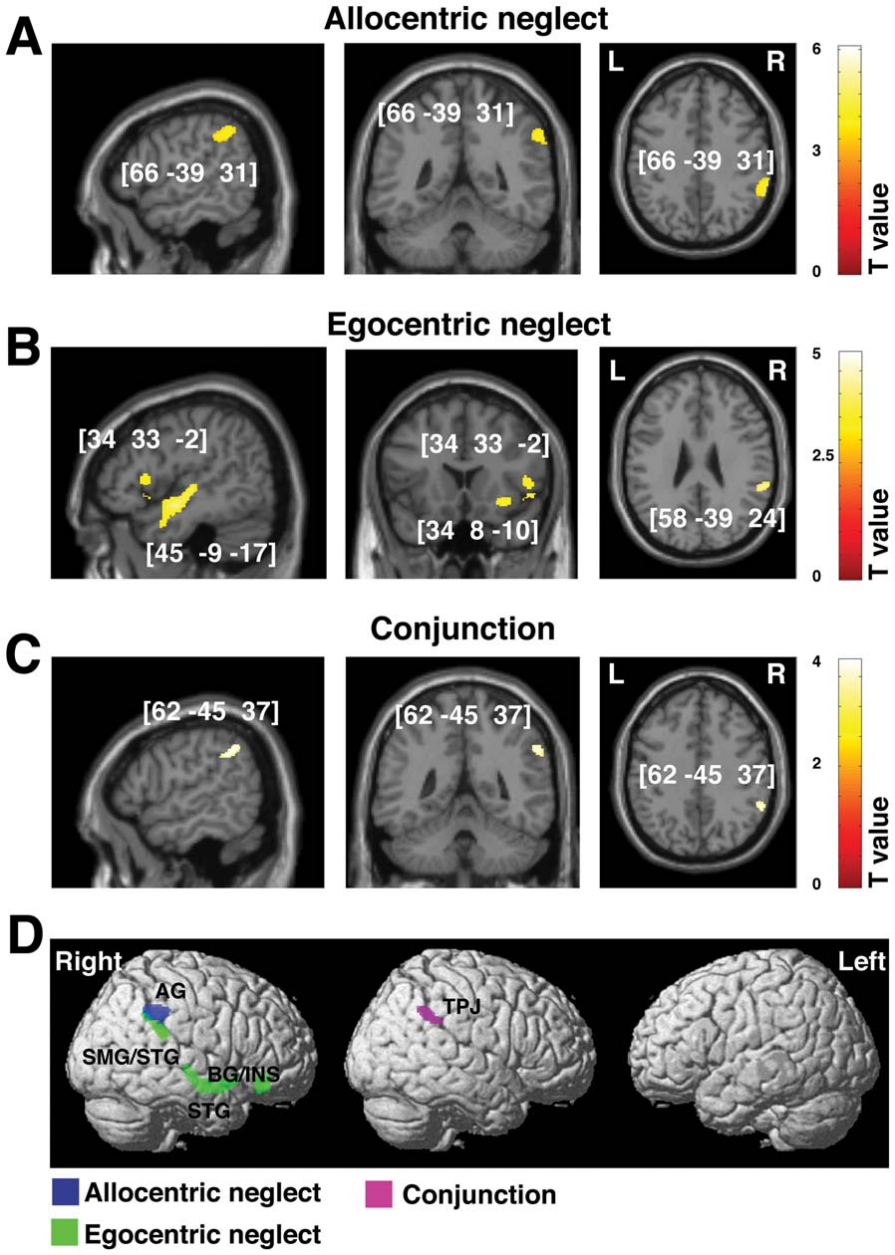
Voxel-wise statistical analysis of grey matter damage: allocentric vs. egocentric neglect at the chronic phase following stroke. VBM results showing voxels corresponding to grey matter damage in (**A**) left allocentric, (**B**) left egocentric and (**C**) both forms of neglect (conjunction analysis). Please note that in **A**, **B** and **C** the lesioned areas are coloured according to their significance level in the VBM analysis, where a brighter colour indicates a higher t-value. The numbers in brackets indicate the peak MNI coordinates. (**D**) To further illustrate the relationship between grey matter loss and any associated allocentric or egocentric symptoms at the chronic phase, all clusters identified by VBM as described above are plotted on a rendered brain. AG, angular gyrus; BG, basal ganglia; INS, insula; SMG, supramarginal gyrus; TPJ, temporal-parietal junction.

We used VBM based on the general linear model to investigate relationship between the grey matter substrates of sub-acute vs. persistent allocentric and egocentric symptoms of visual neglect. We did not observe any reliable results associated with right neglect symptoms. The behavioural data suggest that these symptoms were less frequent than the left neglect symptoms after right hemisphere damage (see Table 1, Figure 1 and Figure S2; this is in agreement with previous reports, for a review see [8]). Therefore, all further reported results concerns only left neglect, which for simplicity is referred to neglect only. The results however showed striking dissociations between the grey matter damage associated with both sub-acute and chronic allocentric neglect and that associated with egocentric neglect (Figure 2A,B and 3A,B; Table 3 and 4). When measured at the sub-acute stage, left allocentric neglect was associated with right hemisphere lesions in frontal regions (the middle and inferior frontal gyri), the inferior parietal lobule partly extending into the superior temporal sulcus, and the middle temporal (partly extending into inferior temporal) and superior occipital gyri (Figure 2A, Table 3). In contrast, sub-acute left egocentric neglect was linked to damage to more anterior parts of the right hemisphere including the middle frontal gyrus, the postcentral gyrus extending into anterior part of supramarginal gyrus, the anterior and central superior temporal gyri and the precuneus (Figure 2B, Table 3).

**Table 3:** Grey matter substrates of sub-acute allocentric vs. egocentric neglect (VBM: Analysis 1).

Contrast	Cluster level		Voxel level		Coordinates	Brain Structure (location)
	P_FWE_	Size	Z-score	Inter* F(1,141)	X Y Z	
**Left allocentric neglect** *						
	0.000	1643	4.73	6.99	**42 −30 24**	Right IPL (SMG and angular gyrus), STS
			4.00		57 −33 39	
	0.000	603	4.36	11.19	**34 30 6**	Right IFG
	0.000	283	4.37	9.62	**21 −78 24**	Right superior occipital gyrus
	0.000	661	4.36	4.90	**56 −33 −15**	Right MTG/ITG
	0.000	254	3.69	5.91	**36 11 34**	Right MFG, sup precentral sulcus
**Left egocentric neglect** *						
	0.000	1980	4.48	8.55	**52 −16 27**	Right postcentral gyrus, SMG
	0.000	334	3.66	9.35	**32 27 30**	Right MFG
	0.000	849	3.68	9.16	**10 −61 33**	Right precuneus
	0.000	527	3.76	4.02	**50 −19 −2**	Right STG
	0.000	302	3.12	10.2	**14 −16 63**	Right SFS
**Common effect (conjunction analysis)**						
	0.001	154	3.76		**45 −24 27**	Right TPJ

Abbreviations: IFG, inferior frontal gyrus; IPL, inferior parietal lobule; ITG, inferior temporal gyrus; MFG, middle frontal gyrus; MTG, middle temporal gyrus; SFS, superior frontal sulcus; SMG, supramarginal gyrus; STG, superior temporal gyrus; STS, superior temporal sulcus; TPJ, temporo-parietal junction; VBM, voxel-based morphometry.

* To further verify the observed dissociations between allocentric and egocentric neglect, we report here the results (F-tests) of the interaction analyses between allocentric and egocentric neglect, these analyses directly test whether brain-behaviour correlations observed for allocnetric neglect are significantly higher than those observed for egocentric neglect, and vice versa.

**Table 4:** Grey matter substrates of chronic allocentric vs. egocentric neglect (VBM: Analysis 2).

Contrast	Cluster level		Voxel level		Coordinates	Brain Structure (location)
	P_FWE_	Size	Z-score	Inter* F(1,141)	X Y Z	
**Left allocentric neglect** *						
	0.000	256	4.19	7.56	**66 −39 31**	Right angular gyrus
**Left egocentric neglect** *						
	0.000	344	4.12	17.25	**34 33 −2**	Right insula
	0.000	330	3.72	13.07	**34 8 −10**	Right insula, BG
	0.000	240	3.69	12.47	**58 −39 24**	Right SMG, STG
	0.000	293	3.65	13.41	**45 −9 −17**	Right STG
**Common effect (conjunction analysis)**						
	0.000	305	4.20		**62 −45 37**	Right TPJ

Abbreviations: SMG, supramarginal gyrus; STG, superior temporal gyrus; TPJ, temporo-parietal junction; VBM, voxel-based morphometry.

* To further verify the observed dissociations between allocentric and egocentric neglect, we report here the results (F-tests) of the interaction analyses between allocentric and egocentric neglect, these analyses directly test whether brain-behaviour correlations observed for allocnetric neglect are significantly higher than those observed for egocentric neglect, and vice versa.

The scans acquired at the sub-acute stage also predicted the substrates of persistent neglect at 9 months. The VBM analyses showed that although widespread lesions were associated with sub-acute neglect symptoms, only damage within a subset of the regions was critically associated with chronic neglect. Specifically, we found that lesions in the right hemisphere within the angular gurus were associated with persistent allocentric symptoms (Figure 3A, Table 4), while lesions within the posterior section superior temporal gyrus at the junction with and extending into the supramarginal gyrus were associated with persistent egocentric neglect (Figure 3B, Table 4). In addition, we found associations between chronic egocentric symptoms and lesioned voxels within the basal ganglia and insula (Table 4). We note that among patients with chronic allocentric neglect, a larger number of patients had both types of neglect deficit (i.e. both egocentric and allocentric symptoms) and a smaller number had pure allocentric neglect. However, our neuroanatomical findings were not due to the larger number of patients with a combination of ego- and allocentric neglect driving performance, since the patients with pure allocentric neglect showed a similar level of severity for allocentric neglect as those with both symptoms (t(9) = 0.34, p = 0.74, 2-tailed, for a comparison of the severity of allocentric neglect for those with only this disorder and for those with both allo- and egocentric neglect). Similarly, there was no difference at the sub-acute stage in the severity of allocentric symptoms between patients with pure allocentric neglect and those with both symptoms (t(20) = −0.16, p = 0.88, 2-tailed). Note that our VBM analysis used continuous measures of neglect and so should be sensitive to neglect severity.

Importantly, our analysis also allowed us to test for substrates that are common for both types of neglect. The conjunction analysis revealed that damage within the right temporo-parietal junction (TPJ) was associated with both left allocentric and left egocentric errors on the Apple Cancellation Task and that lesions within this regions were critical for persistent symptoms (Figure 2C and Figure 3C, Tables 3–4).

### White matter: Acute versus chronic prognosis of allocentric versus egocentric symptoms

**Figure 4:**
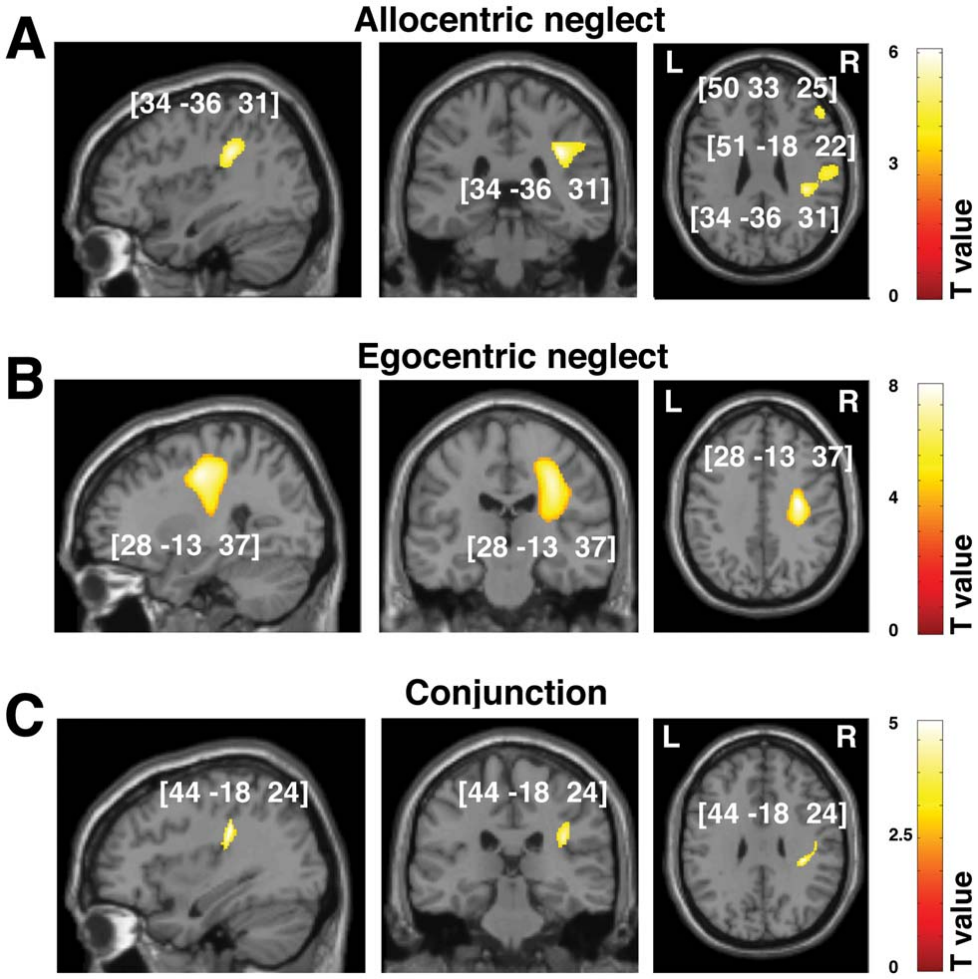
Voxel-wise statistical analysis of white matter damage: allocentric vs. egocentric neglect at the sub-acute phase following stroke. VBM results showing voxels corresponding to white matter damage in (**A**) left allocentric, (**B**) left egocentric and (**C**) both forms of neglect (conjunction analysis). Please note that in **A**, **B** and **C** the lesioned areas are coloured according to their significance level in the VBM analysis, where a brighter colour indicates a higher t-value. The numbers in brackets indicate the peak MNI coordinates.

**Figure 5:**
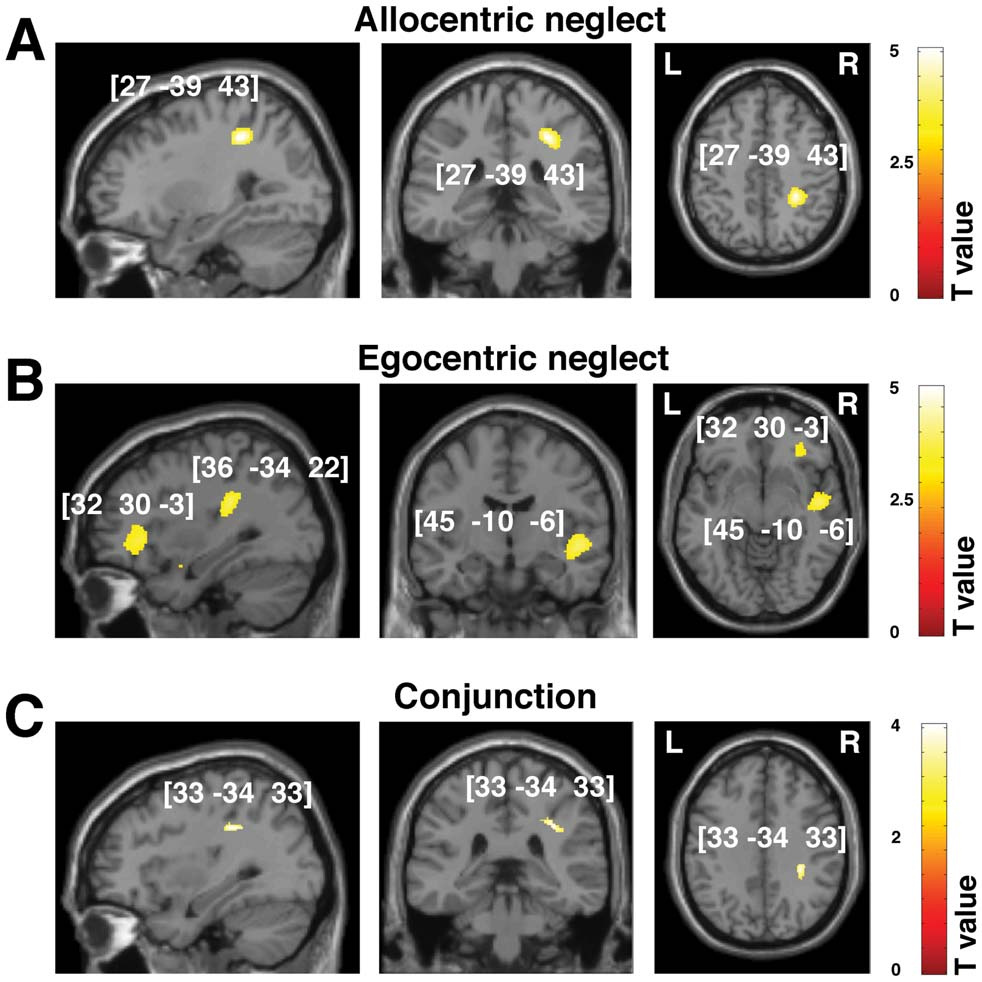
Voxel-wise statistical analysis of white matter damage: allocentric vs. egocentric neglect at the chronic phase following stroke. VBM results showing voxels corresponding to white matter damage in (**A**) left allocentric, (**B**) left egocentric neglect and (**C**) both forms of neglect (conjunction analysis). Please note that in **A, B** and **C** the lesioned areas are coloured according to their significance level in the VBM analysis, where a brighter colour indicates a higher t-value. The numbers in brackets indicate the peak MNI coordinates.

Similar to the assessments of grey matter damage we used VBM analyses to co-vary out allocentric and egocentric components of visual neglect at the sub-acute and chronic phases following stroke. Again, we did not observed any reliable results for the right neglect symptoms, findings are therefore reported only for left neglect. The analyses demonstrated that disconnections resulting from damage along the right superior longitudinal fasciculus (SLF) were associated with both types of neglect symptoms and related to both sub-acute and chronic deficits (Tables 5–6, Figure 4 and 5). This was further confirmed by VBM-based conjunction analyses (Tables 5–6). Furthermore, we showed that damage within the anterior part of the inferior fronto-occipital fasciculus (IFOF) and the uncinate fasciculus were associated with both types of neglect symptoms, while damage within the inferior longitudinal fasciculus (ILF), superior corona radiata and superior thalamic radiations was associated with egocentric neglect. Disconnections within these additional long association pathways were critical for both sub-acute and chronic neglect symptoms (Tables 5–6; Figure 4 and 5).

**Table 5:** White matter substrates of sub-acute allocentric vs. egocentric neglect (VBM: Analysis 1).

Contrast	Cluster level		Voxel level		Coordinates	Brain Structure (location)
	P_FWE_	Size	Z-score	Inter* F(1,142)	X Y Z	
**Left allocentric neglect** *						
	0.000	2142	5.09	24.34	**34 −36 31**	Right SLF
			4.12		51 −18 22	
	0.000	703	4.09	4.73	**50 33 25**	Right IFOF, UNC
			4.00		60 18 10	
**Left egocentric neglect** *						
	0.000	3723	7.02	58.68	**28 −13 37**	Right SLF, sup CR; sup TR
	0.000	186	3.47	12.79	**16 45 3**	Right IFOF, UNC
**Common effect (conjunction analysis)**						
	0.000	291	4.00		**44 −18 24**	Right SLF

Abbreviations: IFOF, inferior fronto-occipital fasciculus; SLF, superior longitudinal fasciculus; sup, superior; TR, thalamic radiation; UNC, uncinate fasciculus; VBM, voxel-based morphometry.

* To further verify the observed dissociations between allocentric and egocentric neglect, we report here the results (F-tests) of the interaction analyses between allocentric and egocentric neglect, these analyses directly test whether brain-behaviour correlations observed for allocnetric neglect are significantly higher than those observed for egocentric neglect, and vice versa.

**Table 6:** White matter substrates of chronic allocentric vs. egocentric neglect (VBM: Analysis 2).

Contrast	Cluster level		Voxel level		Coordinates	Brain Structure (location)
	P_FWE_	Size	Z-score	Inter* F(1,142)	X Y Z	
**Left allocentric neglect** *						
	0.000	629	4.76	18.57	**27 −39 43**	Right IFOF, SLF
**Left egocentric neglect** *						
	0.000	662	4.00	15.05	**32 30 −3**	Right IFOF, UNC
	0.000	988	4.10	14.85	**45 −10 −6**	Right ILF, SLF
			3.98		50 −10 −6	
	0.000	1447	4.84	26.20	**36 −34 22**	Right SLF, post TR
**Common effect (conjunction analysis)**						
	0.000	121	3.38		**33 −34 33**	Right SLF

Abbreviations: IFOF, inferior fronto-occipital fasciculus; ILF, inferior longitudinal fasciculus; post, posterior; SLF, superior longitudinal fasciculus; TR, thalamic radiation; UNC, uncinate fasciculus; VBM, voxel-based morphometry.

* To further verify the observed dissociations between allocentric and egocentric neglect, we report here the results (F-tests) of the interaction analyses between allocentric and egocentric neglect, these analyses directly test whether brain-behaviour correlations observed for allocentric neglect are significantly higher than those observed for egocentric neglect, and vice versa.

### Lesion volume and neglect symptoms

Overall lesion size (volume) in the neglect group was not significantly larger than in the group of patients without neglect symptoms (t(158) = 1.7, p>0.5; see Table 1). However, we found significant difference in the lesion volume between patients who recovered from neglect and patients with chronic deficits (all types of neglect symptoms t(51) = −3.44; p<0.001; left deficits only t(36) = −2.76; p<0.01). We next tested whether there was a relationship between the severity of left (either allocentric or egocentric) neglect symptoms and lesion volume. We found no significant correlations between lesion volume and the severity of either sub-acute left allocentric neglect (r = 0.15, p = 0.36) or left egocentric neglect (r = 0.21, p = 0.20). Importantly lesion volume was not a significant predictor of recovery rate of either left allocentric (r = 0.13; p = 0.41) or left egocentric (−0.22 p = 0.20) neglect (correlation between lesion volume and the difference in performance at chronic and sub-acute phase). Finally, although the correlation between the severity of sub-acute and chronic left allocentric neglect(r = 0.29; p = 0.08) and the correlation between the severity of sub-acute and chronic left egocentric (r = 0.32; p = 0.06) neglect were not significant, they show clear trend and the lack of significance was likely due to low variability across the sample as roughly half of the patients had no sign of neglect at the sub-acute stage.

## Discussion

The current study examined whether information gained from computed tomography scans acquired as a part of routine clinical diagnosis following stroke has the potential to predict recovery vs. persistent symptoms associated with visuospatial neglect. Our data support a dissociative account of egocentric and allocentric neglect both in terms of the distinct behavioural deficits and the associated neuronal substrates [9], [14], [26], [27]. Importantly, the findings also indicate that the substrates of persistent neglect can be predicted from clinical scans acquired sub-acutely following stroke. We showed that lesions in the angular gyrus were associated with persistent allocentric symptoms, while lesions within the superior temporal gyrus extending into the supramarginal gyrus, as well as damage to the basal ganglia and insula, were associated with persistent egocentric neglect. Furthermore, we found that that damage within temporo-parietal junction (TPJ) and white matter disconnections resulting from damage along the superior longitudinal fasciculus were critically linked to the persistent presence of both types of neglect. Bickerton et al. [10] reported that patients with both types of neglect tended to have a worse functional outcome at 9 months than patients with only egocentric or only allocentric neglect, and that the presence of both sets of symptoms was additionally linked to the presence of depression. The present analysis suggests that the poor outcomes are linked to the presence of damage to the right TPJ as well as proximal white matter.

These findings are in direct agreement with our previous work into the neural correlates of allocentric and egocentric errors on the Apple Cancellation Task in chronic brain injured patients scanned using MRI [27]. Specifically, both analyses point to damage to chronic allocentric problems being linked to the angular gyrus while chronic egocentric symptoms are associated with damage within the supramarginal and superior temporal gyri and the basal ganglia. Yet again, this work supports the argument that distinct cortical regions control attention across (egocentric) space and (allocentric) attention within objects (‘between’ and ‘within object’ spatial representations; see [67]. In addition, some common cortical regions (mainly the right TPJ), and common white matter pathways (mainly the SLF), support attention to both spatial and object-based representations (see [27]). Our findings are also in agreement with these by Silvetti et al. [85] based on neuropsychological data combined with neural network stimulations indicating the link between allocentric coding frame and the dorsal-parietal-frontal network.

Alternative accounts of the distinction between egocentric and allocentric neglect can also be offered. One is that egocentric neglect reflects a problem in global space perception while allocentric neglect reflects a problem in representing space at a more local scale. Halligan and Marshall [86] proposed that left neglect after right hemisphere damage is brought about by the combination of poor global space perception along with a spatial bias in attention. In the Apples test of neglect, poor global perception could lead to patients not attending to one side of the page. Poor attention to local spatial areas is associated with left rather than right hemisphere damage [68] and, if coupled to a spatial bias in selection, then there may be poor detection of missing parts on one side of individual objects – what we have labelled as allocentric neglect. However we found no evidence that allocentric neglect was particularly associated with left hemisphere damage, as might be expected on this account. In addition, the Apples test uses both large and small apples, which may correspond to global and more local representations, but there was no evidence for any bias based on the sizes of the stimuli. A further possibility is that both forms of neglect stem from a gradient of attention across egocentric space (e.g., [24]. On this gradient account, there will be a bias against elements on one side of objects, even when the objects fall in the ipsilesional visual field. Again, this account has problems with the data. For example, it predicts that allocentric and egocentric neglect should co-occur behaviourally and they should be underpinned by common lesion sites. In contrast to this the behavioural data here indicate dissociations between patients with one or other form of neglect and, in addition, egocentric and allocentric neglect are associated with contrasting lesions. This gradient account also fails to explain prior results where opposite egocentric and allocentric biases have occurred even in the same patient, which also arose in some cases in the present sample [22], [23].

The current study demonstrated that the anatomical distinctions between the different forms of neglect arose not only at the sub-acute phase but also at the chronic phase following stroke. This matches the data from prior imaging studies undertaken at the chronic stage after brain injury [27]. However, it should be also noted that, in comparison to our previous work, the current study identified a more confined network of cortical and sub-cortical regions associated with chronic neglect. This could be explained by the fact that, in contrast to Chechlacz et al. (2010), the neural substrates of chronic deficits were examined here using scans acquired at a sub-acute phase. Consequently, we were unable to take into account additional brain damage in chronic cases, which may result from secondary infarcts/degeneration in cortical regions that were initially structurally intact but affected by perfusion abnormalities [69], [70], [71], [72]. Thus, if anything, our analyses provide an underestimation of the contribution of sub-acute lesions to chronic neglect symptoms.

Persistent neglect is associated with overall poor functional outcome following stroke [1], [10], [46]. Previous work suggests that the initial severity of deficits, the presence of visual field defects, the age at which the lesion occurred and the presence of age-associated brain atrophy are useful indicators of recovery in addition to lesion size and location [4], [43], [44], [45], [46], [47], [48], [49], [80]. The current study however shows that lesion location alone can serve as a critical predictor for persistent neglect symptoms even when the other factors are co-varied out in the analysis. This sets our study apart from the previous work (e.g., [50]) where only the severity of neglect has been controlled. We also note a clear trend in the correlation between the severity of sub-acute and chronic neglect symptoms, for each type of neglect, indicating that the severity of behavioural symptoms might be a useful predictor of recovery in the absence of neuroimaging findings on clinically obtained CT scans.

Importantly, as discussed above, our study takes into account the dissociation between neglect symptoms and emphasizes the neural substrates underlying recovery of the different symptoms. For example Saj et al. [52] demonstrated that pooling across different measures (tests) for neglect, without differentiating between the varied symptoms, will highlight the more frequent sites of damage, but these will not necessarily be causally related to specific symptoms. A final point with regards to lesion location and lesion size should be addressed here. In the studied sample we included both MCA and PCA stroke patients as well as patients with other types of stroke in order to generalize our findings to the entire clinical populations showing neglect. However, it should be noted that previous work indicates that strokes affecting these two different cerebral artery territories have been shown to result in different lesion size and have been associated with neglect symptoms linked to different lesion locations (for further comprehensive discussion see [30]). In the current study we have not contrasted patients with different types of stroke but our analyses examined the link between lesion volume and neglect symptoms. Although, there was no significant difference in the lesion volume between patients with and without neglect, importantly we found a significant difference in the lesion volume between patients who recovered from neglect and patients with persistent deficits. However, neither lesion volume nor the initial severity of symptoms was a significant predictor of either left allocentric or left egocentric chronic neglect severity.

Samuelson et al. [48] first reported that lesions in deep white matter were highly correlated with persistent neglect. Their findings related chronic symptoms to paraventricular white matter in the temporal lobe. These results are consistent with our data showing the link between damage within ILF and persistent egocentric neglect as well as reported here strong overall association between chronic neglect symptoms and damage within white matter pathways. It should be noted though that the link between the ILF and egocentric neglect found here is not consistent with recent neglect studies implicating the critical role of parieto-frontal white matter pathways (see below) although damage within this fasciculus has been previously reported in neglect patients [27], [41].

Importantly, our white matter analyses indicated that damage within the SLF, in addition to other long association pathways, was critically associated with both persistent allocentric and egocentric neglect symptoms. The SLF is the main component of the attention network connecting temporo-parietal association areas with the frontal lobes [36], [73], [74], [75]. The link between the SLF and neglect is consistent with an interpretation of the syndrome as deriving from parieto-frontal disconnection (for extensive review see [36], also such interpretation lies in agreement with pioneering earlier reports from both monkeys [87] and human [88]). Other recent studies link white matter disconnections resulting from damage within SLF to unilateral neglect [27], [37], [38], [39], but without showing that damage at the sub-acute stage predicts longer-term recovery, as we do here. The current evidence is consistent with the view that separate egocentric (between-object) and allocentric (within-object) representations in the cortex link through common pathways to frontal brain regions concerned with action [67], [76].

### Conclusions and methodological considerations

The present paper shows that it is possible to conduct lesion-symptom mapping using clinically-acquired CT scans, and this can complement research-based scanning using high-resolution MRI. Specifically, the current findings are in direct agreement with our previous study examining (in a different sample of chronic brain injured patients) neural correlates of allocentric and egocentric neglect based on high resolution structural and diffusion scans [27]. This indicates that it may be possible to use clinical scans to predict outcome for individual patients. There are however some potential limitations. First, it is known that lesions resulting from ischemic stroke may be underestimated when CT scans are taken early-on after a stroke [77]. Secondly, CT scans fail to detect cortical dysfunction within a region that is structurally intact but has inadequate cortical perfusion, and this dysfunction may contribute to cognitive deficits. The second point is particularly critical as previous reports have linked cortical malperfusion at acute/sub-acute stroke to deficits in spatial attention [14], [33], [78], [79] and reperfusion of cortical areas within 3–5 days following stroke to improvement of neglect symptoms [53]. It should be noted that although CT scans do not have the quality or resolution provided by MRI, these are routinely used for stroke diagnosis. Despite the limitations, though, the current study indicates that the site of damage, revealed by semi-automated analysis of clinical scans, can help predict the long-term presence of different forms of unilateral neglect. We conclude that the presented findings strongly implicate the potential of using CT data to predict functional recovery (both in terms of lesion location and lesion volume) and we advocate that the use of this imaging modality to develop novel tools for making clinically meaningful predictions of stroke outcome based on (for example) machine learning approaches.

Another important methodological point should be made here with reference to the voxel-wise analysis of the white matter lesions. Neuronal fibers may be damaged at several different points along the white matter tract and yet this may result in the same functional outcome i.e. behavioural deficits, suggesting that voxel-based approach may be too conservative for assessing disconnection problems. In the current study we combined the modified tissue segmentation protocol with a voxel-based morphometry approach in order to look for common structure-function relationships separately for grey and white matter and we then localized white matter lesions associated with different visuospatial problems to specific white matter pathways. While this might be a conservative way to assess for white matter disconnections, it does at least take a common approach to examining both grey and white matter substrates of cognitive deficits.

The final methodological concern is linked to the diagnosis of neglect. In contrast to previous reports examining the neuroanatomy of neglect, including recent reports examining neglect recovery [50], [52], we used a single task that simultaneously measured both allocentric and egocentric symptoms. The comprehensive diagnosis of neglect benefits from the use of multiple tests, as this allows us to detect heterogeneous neglect symptoms – including those that may otherwise go undetected in some patients (e.g. [89], [52]). Moreover, pooling the data across multiple tests can mask dissociative symptoms (for recent review see [90], [91]). Our intension was to use a single task that simultaneously measures both allocentric and egocentric symptoms as this allowed us to control for variability in patients' behaviour due to differential task demands, test conditions and stimuli that could potentially arise when using different tests. Our data support a recent ALE meta-analysis which showed high concurrence in the neural substrates of egocentric and allocentric neglect across a variety of different tests [91]. We conclude that it is the cognitive process that the test measures, and not the test itself, that is critical for revealing neuronal dissociations.

## Supporting Information

Figure S1 **Modified unified segmentation.** (**A**) T1 standard brain, GM (grey matter), WM (white matter) and CSF (cerebrospinal fluid) priors from SPM8. (B) Examples of output of the modified unified segmentation of patients' CT scans.
(TIF)

Figure S2 **Frequency of allocentric and egocentric neglect after right brain damage (RBD), left brain damage (LBD) and in patients with no visible lesions on CT scans (NVL); contra = contralesional symptoms; ipsi = ipsilesional symptoms; left = left deficits; right = right deficits.**
(TIF)

Figure S3 **Lesion distribution.** Lesion overlap map representing the spatial distribution of lesions in 160 patients included in the study. Lesion maps from individual patients were reconstructed based on method described in Methods S1. The lesion overlap map is shown for seven axial slices in standard MNI space. The colour bar represents the number of patients with a lesion within particular voxel (range 1–160). MNI Z-coordinates of the axial sections are given.
(TIF)

Figure S4 **Lesion reconstruction.** (**A–E**) Examples of lesion reconstructions for 5 patients from the current study, including example of smaller (A) versus larger (C) ischemic strokes, subcortical lesions (D,E) and hemorrhagic stroke with secondary infarct (D). CT = normalized CT scan; GM = segmented grey matter; WM = segmented white matter; Lesion = binary lesion map overlaid on normalized CT scan.
(TIF)

Methods S1 **Lesion reconstruction.** Voxel-based outlier detection procedure based on general linear model.
(DOCX)
