# Supplementary figures and images for: The Prognosis of Allocentric and Egocentric Neglect: Evidence from Clinical Scans
Source: PLoS One. 2012 Nov 1;7(11):e47821. doi: 10.1371/journal.pone.0047821 (PMC3486857; doi:10.1371/journal.pone.0047821)

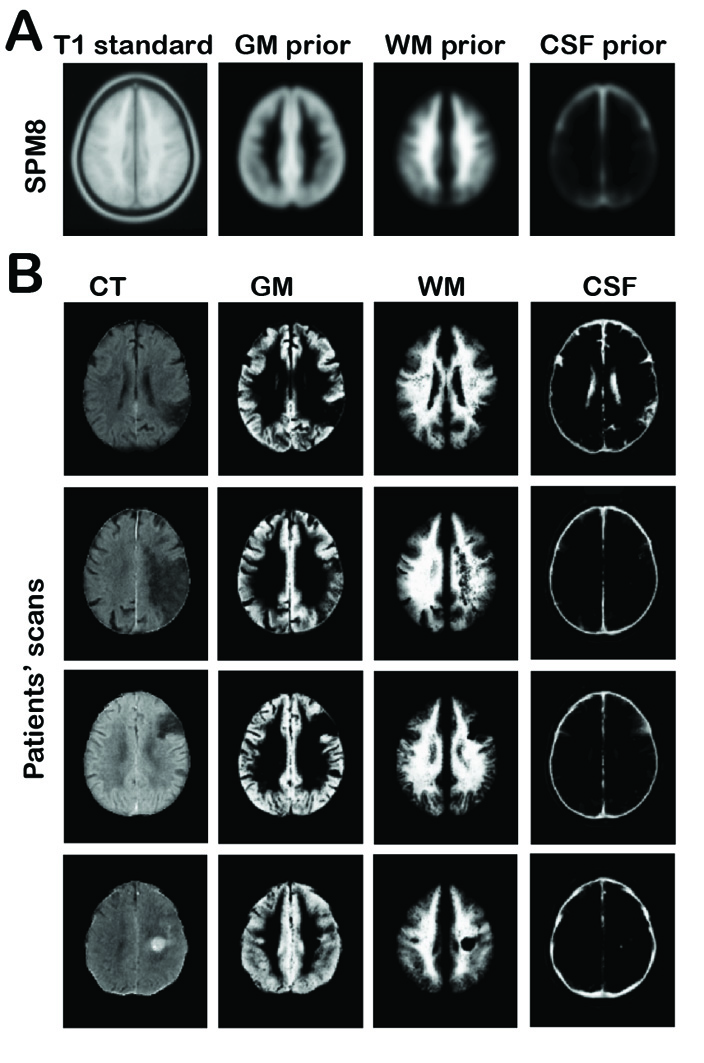

Supplement: Figure S1 — Modified unified segmentation. (A) T1 standard brain, GM (grey matter), WM (white matter) and CSF (cerebrospinal fluid) priors from SPM8. (B) Examples of output of the modified unified segmentation of patients' CT scans. (TIF) [file pone.0047821.s001.tif]

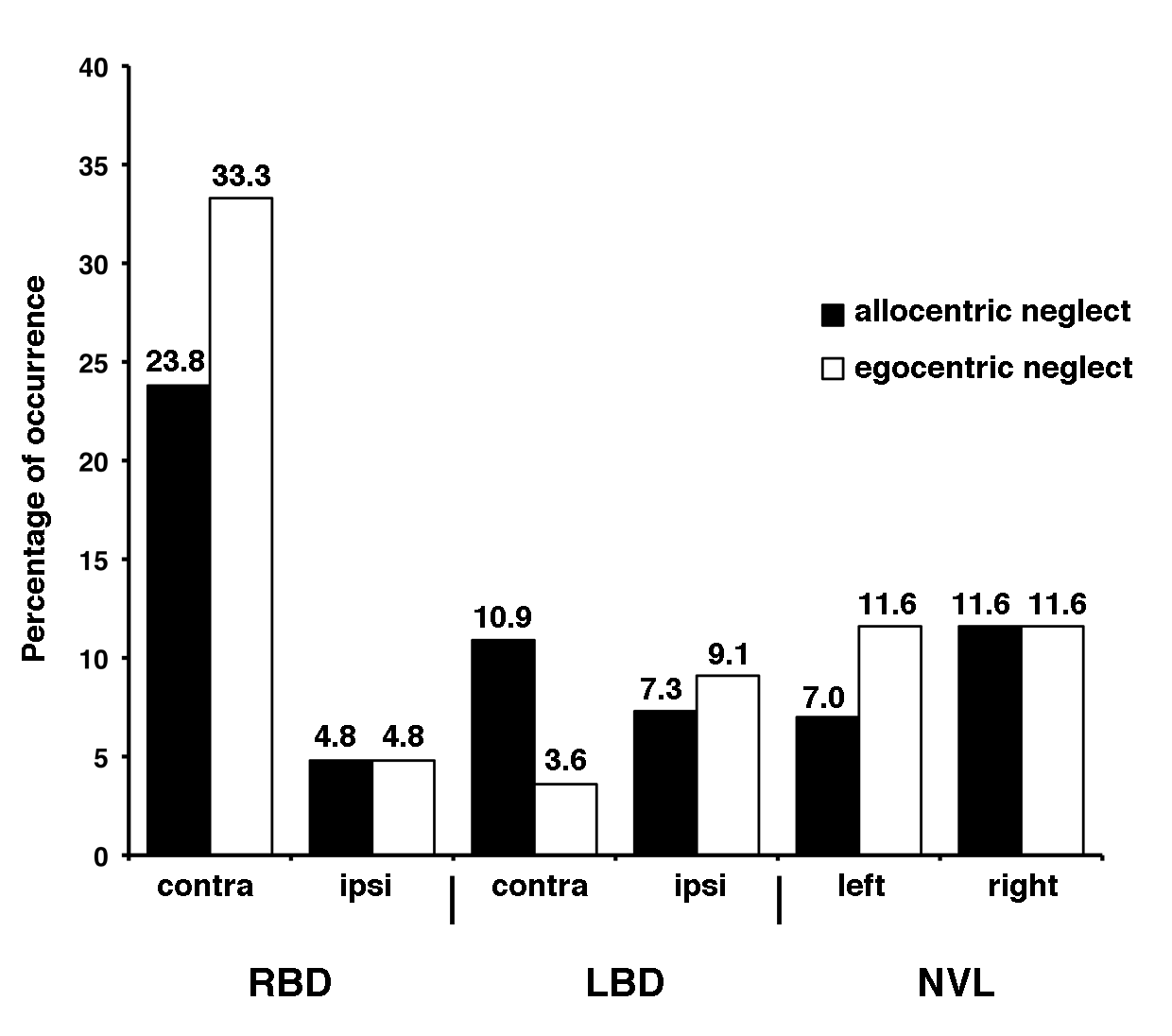

Supplement: Figure S2 — Frequency of allocentric and egocentric neglect after right brain damage (RBD), left brain damage (LBD) and in patients with no visible lesions on CT scans (NVL); contra = contralesional symptoms; ipsi = ipsilesional symptoms; left = left deficits; right = right deficits. (TIF) [file pone.0047821.s002.tif]

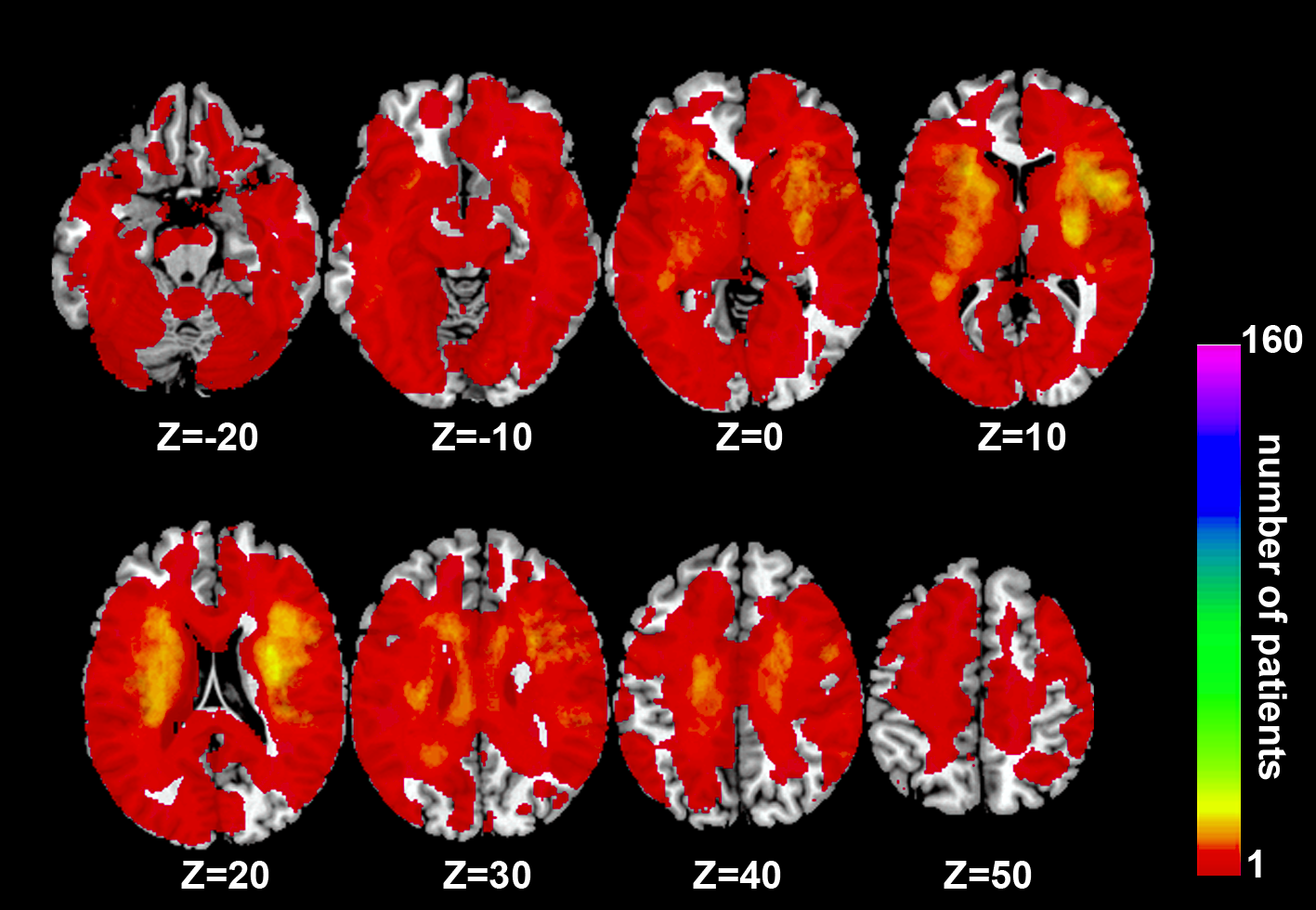

Supplement: Figure S3 — Lesion distribution. Lesion overlap map representing the spatial distribution of lesions in 160 patients included in the study. Lesion maps from individual patients were reconstructed based on method described in Methods S1. The lesion overlap map is shown for seven axial slices in standard MNI space. The colour bar represents the number of patients with a lesion within particular voxel (range 1–160). MNI Z-coordinates of the axial sections are given. (TIF) [file pone.0047821.s003.tif]

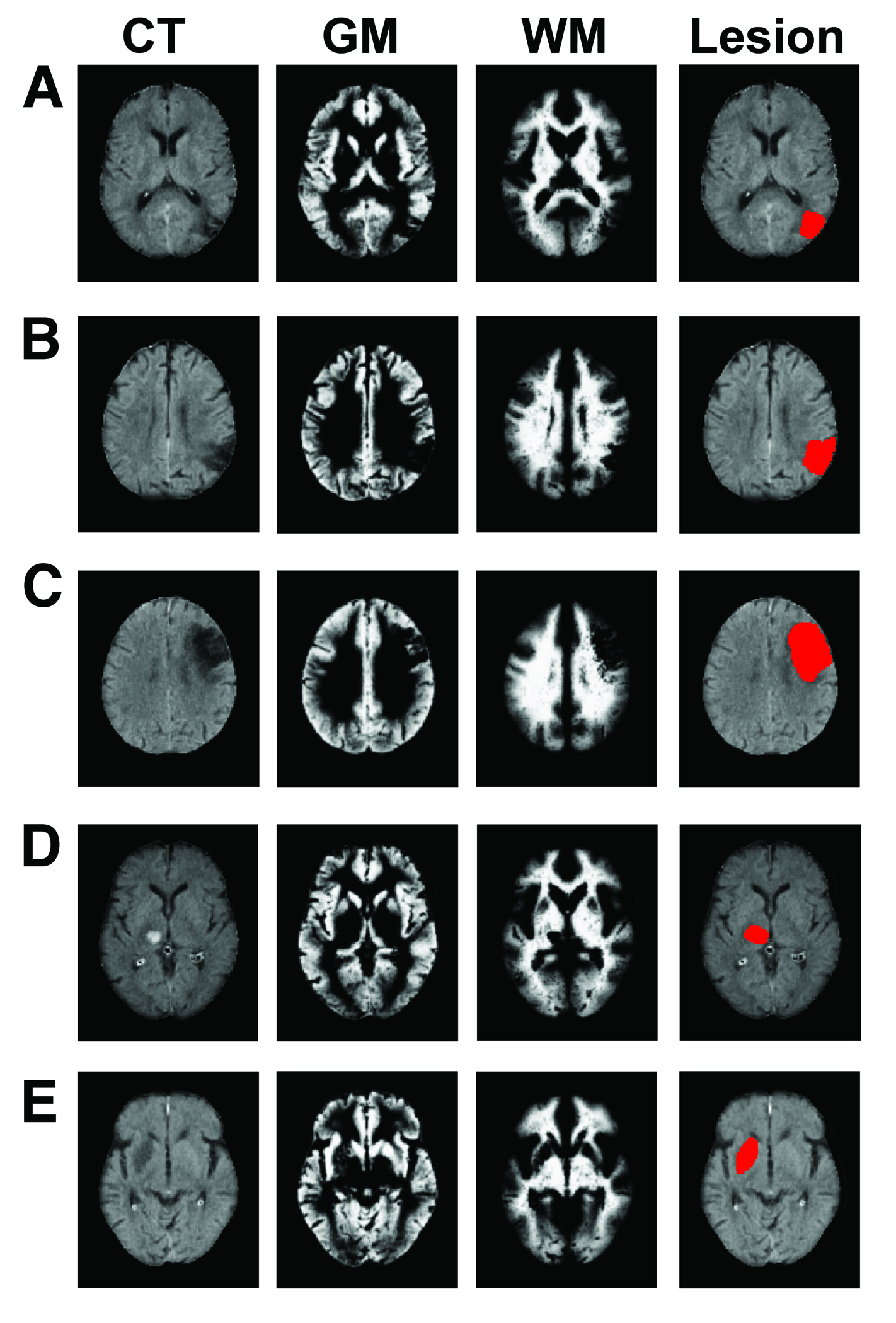

Supplement: Figure S4 — Lesion reconstruction. (A–E) Examples of lesion reconstructions for 5 patients from the current study, including example of smaller (A) versus larger (C) ischemic strokes, subcortical lesions (D,E) and hemorrhagic stroke with secondary infarct (D). CT = normalized CT scan; GM = segmented grey matter; WM = segmented white matter; Lesion = binary lesion map overlaid on normalized CT scan. (TIF) [file pone.0047821.s004.tif]
