# Supplementary material for: The Prognosis of Allocentric and Egocentric Neglect: Evidence from Clinical Scans
Source: PLoS One. 2012 Nov 1;7(11):e47821. doi: 10.1371/journal.pone.0047821 (PMC3486857; doi:10.1371/journal.pone.0047821)
Supplement: Methods S1 — Lesion reconstruction. Voxel-based outlier detection procedure based on general linear model. (DOCX) [file pone.0047821.s005.docx]

**Supplementary Methods S1**

***Lesion reconstruction.*** The lesion of each patient included in the current study was semi-automatically identified using a modified unified segmentation (as described in the Methods section) and whole brain voxel-based outlier detection based on general linear model. Tissue abnormalities (lesions) for all patients were first reconstructed from CT scans using a voxel-based analysis with SPM8 (Statistical Parametric Mapping, Welcome Department of Cognitive Neurology, London UK) by comparing each patient’s segmented GM and segmented WM to the segmented GM and segmented WM of 105 scans of brain with no visible lesions. These scans were extracted from our entire CT database that contained more than 600 brain images, hence equating for scanner and various sequence parameters. Computed tomography (CT) scans involve large dose of radiation and thus it would not be justifiable to obtain such scans from “healthy controls” i.e. without medical reason for such brain imaging intervention. Therefore, for the purpose of the current study, we used as control images, CT scans from patients without visible brain lesion. The search using such criteria identified 105 scans from our data base, these belong to 60 males and 45 females; mean age 67, range 29 to 89 years.

In order to delineate brain abnormalities (lesions) we used a GLM and compared each patient to this group while age and gender were modelled as covariates. This first step allowed us to generate the GM and WM outlier maps that coded the degree of abnormality of each voxel (based on the comparison to the 105 scans with no visible lesion). The outlier GM and WM maps were next individually thresholded by verifying against each patient’s normalized CT scan, then combined and binarized to generate lesion map for each individual patient (Figure S4). This simply ‘automates’ and standardises the procedure of a manual delineation of an abnormal tissue by a human observer. The reconstructed lesion maps were further used to calculated lesion volume and to examine spatial distribution of lesions in the studied group of patients.
